# A phase 2, open-label study of brentuximab vedotin in patients with CD30-expressing solid tumors

**DOI:** 10.1007/s10637-019-00768-6

**Published:** 2019-04-16

**Authors:** Jeffrey P. Sharman, Jennifer J. Wheler, Lawrence Einhorn, Afshin Dowlati, Geoffrey I. Shapiro, John Hilton, John M. Burke, Tanya Siddiqi, Nancy Whiting, Shadia I. Jalal

**Affiliations:** 1 Willamette Valley Cancer Institute and Research Center/US Oncology Research, 520 Country Club Rd., Eugene, OR 97401 USA; 2 US Oncology Research, Houston, TX USA; 3 The University of Texas MD Anderson Cancer Center, 1515 Holcombe Blvd., Houston, TX 77030 USA; 4 Indiana University Division of Hematology and Oncology, 535 Barnhill Dr, Indianapolis, IN 46202 USA; 5 Department of Medicine – Hematology and Oncology, University Hospitals Case Medical Center, 11100 Euclid Ave., Cleveland, OH 44106 USA; 6 Dana-Farber Cancer Institute, 450 Brookline Ave., Boston, MA 02215 USA; 7 Department of Medicine, Division of Medical Oncology, The Ottawa Hospital and University of Ottawa, 501 Smyth Road, Ottawa, Ontario K1H 8L6 Canada; 8 Ottawa Hospital Research Institute, 501 Smyth, Box 511, Ottawa, Ontario K1H 8L6 Canada; 9 Rocky Mountain Cancer Centers, 1700 South Potomac St., Aurora, CO 80012 USA; 10 City of Hope National Medical Center, 1500 East Duarte Rd., Duarte, CA 91010 USA; 11 Seattle Genetics, Inc., 21823 30th Dr. SE, Bothell, WA 98021 USA

**Keywords:** CD-30, Solid tumors, Brentuximab vedotin, Antibody-drug conjugate

## Abstract

*Purpose* Brentuximab vedotin (BV) is an anti-CD30 antibody-drug conjugate used in the treatment of several types of lymphomas. Expression of the target antigen has also been reported on a variety of malignant tumors of nonlymphoid origin. This phase 2, open-label study evaluated the safety and antitumor activity of BV in patients with CD30-expressing nonlymphomatous malignancies. *Methods* Patients were dosed with 1.8 or 2.4 mg/kg BV once every three weeks. Antitumor activity was assessed at Cycles 2, 4, and every 4 cycles thereafter. Patients with stable disease or better were eligible to continue treatment until disease progression, unacceptable toxicity, or study closure. *Results* Of the 2693 patients screened, 3.8% had solid tumors with CD30 expression and 63 eligible patients with solid tumors enrolled in this study. The most common CD30 positive solid tumors were testicular cancer and mesothelioma. Both subtypes had more than one patient with an objective response. The median duration of BV exposure was 6.1 weeks. The disease control rate, defined as achieving stable disease or better at any point during the study, was 55%. The objective response rate was 11%, with a median duration of response of 2.92 months. The most common adverse events reported were fatigue (57%), nausea (33%), and decreased appetite (32%). *Conclusion* The safety profile of BV in patients with solid tumors was similar to the known safety profile of BV. In solid tumors, BV had modest activity as a single agent, which was similar to other second-line treatments already available to patients.

**Electronic supplementary material:**

The online version of this article (10.1007/s10637-019-00768-6) contains supplementary material, which is available to authorized users.

## Introduction

CD30 is a transmembrane glycoprotein belonging to the tumor necrosis factor superfamily. It is generally absent or expressed at very low levels in healthy tissues, with the exception of a small subset of normal activated or proliferating B and T lymphocytes [1]. Upon ligand stimulation, CD30 activation leads to pleiotropic effects on cells and tissues that are dependent upon the cell type, differentiation stage, transformation status, and other stimuli present [2]. These effects include induction of proliferation and survival in some cell types and apoptosis and cell death in others [2, 3].

Brentuximab vedotin (ADCETRIS®, Seattle Genetics, Inc., Bothell, WA), an anti-CD30 antibody-drug conjugate (ADC), consists of the chimeric IgG_1_ antibody cAC10, specific to human CD30, covalently attached to the microtubule-disrupting agent monomethyl auristatin E (MMAE) by a protease-cleavable linker. The mechanism of action of brentuximab vedotin involves binding of the ADC to CD30-expressing cells, leading to internalization of the ADC-CD30 complex and the release of MMAE via proteolytic cleavage within the cell. Binding of MMAE to tubulin disrupts the microtubule network within the cell, inducing cell cycle arrest and apoptotic death of the cell [4]. While targeted delivery of MMAE to CD30-expressing cells is the primary mechanism of action of brentuximab vedotin [5], antibody-dependent cellular phagocytosis, immunogenic cell death, and the bystander effect are additional proposed mechanisms of tumor killing that may contribute to the clinical activity of brentuximab vedotin [6–12]. The safety and efficacy of brentuximab vedotin has been shown through its approved use in treating stage III/IV classical Hodgkin lymphoma, relapsed or refractory anaplastic large cell lymphoma, and previously untreated CD30-expressing PTCL [13].

Expression of CD30 has also been reported on malignant tumors of nonlymphoid origin, including testicular embryonal carcinoma [14–16], lung adenocarcinoma and mesothelioma [17], mesenchymal tumors [18], granulocytic sarcoma [19], mastocytosis [20], and acute myelogenous leukemia [21]. The absence of CD30 expression in healthy nonlymphoid tissues and its observed expression in several nonlymphoid malignancies may make it a potential therapeutic target in nonlymphomatous malignancies.

The current phase 2, open-label study (NCT01461538), sponsored by Seattle Genetics, Inc., aimed to evaluate the antitumor activity, safety, immunogenicity, and pharmacokinetics (PK) of brentuximab vedotin in patients with CD30-expressing nonlymphomatous malignancies, with a focus on solid tumors.

## Patients and methods

### Patient eligibility

Eligible patients were 12 years of age or older (or ≥ 6 years of age with permission from the sponsor) with histologically confirmed CD30-expressing nonlymphomatous cancer. Eligible patients’ tumors were screened for CD30 expression in a companion screening protocol (SGN00–001). For solid tumors, CD30 expression was assessed by immunohistochemistry with an anti-CD30 antibody (BerH2) [22] using a cutoff for positivity of 10%. Patients with CD30-expressing disease by central or local pathology review were eligible for enrollment. Measurable disease, defined for solid tumors as ≥1 nonresectable lesion at least 10 mm in the longest diameter, was also required. Patients must have failed, refused, or been deemed ineligible for standard therapy and must have had an Eastern Cooperative Oncology Group (ECOG) Performance Status score of 0 or 1 or a Karnofsky or Lansky Performance Status score of ≥70. Patients with a primary diagnosis of lymphoma or central nervous system malignancy; those with a history of another primary invasive malignancy that had not been definitively treated or in remission for at least 3 years; and those with a documented history of progressive multifocal leukencephalopathy, a cerebral vascular event, unstable angina, myocardial infarction, or cardiac symptoms consistent with New York Heart Association Class III-IV within 6 months prior to the first dose of brentuximab vedotin were excluded. Patients may not have been treated with chemotherapy, radiotherapy, biologics, or other immunotherapies within 4 weeks prior to the first dose of study drug, or with any previous anti-CD30 directed therapy. Patients with allogeneic stem cell transplant within 100 days prior to study start or with graft versus host disease were excluded. Current therapy with other systemic antineoplastic or investigational agents was also a reason for study exclusion.

### Study design and treatment

All study procedures were conducted at investigational sites following approval by Investigational Review Boards. All patients provided written informed consent prior to participation in the study and patients did not receive compensation for their participation.

In patients with solid tumors, dosing was initiated at 1.8 mg/kg Q3W via intravenous infusion over a period of 30 min. During the course of the study, the study protocol was amended to allow dosing at 2.4 mg/kg Q3W in patients with solid tumors. Those subjects originally dosed at 1.8 mg/kg Q3W may have had their dose increased to 2.4 mg/kg Q3W following consultation with the sponsor’s medical monitor. Dose reductions for tolerability were also permitted at both dose levels.

### Study assessments

Response assessments for patients with solid tumors consisted of computed tomography (CT) scans performed at Cycles 2, 4, and every 4 cycles thereafter while the patient was receiving study treatment. Antitumor activity was assessed by investigators based on radiographic tumor imaging according to the Response Criteria for Solid Tumors (RECIST) version 1.1 [23]. Patients with stable disease (SD) or better were eligible to continue treatment until disease progression, unacceptable toxicity, or study closure.

Each safety assessment consisted of an evaluation for adverse events, physical examination, ECOG status, and laboratory evaluations. These were conducted prior to Cycle 1 of therapy and with each cycle of treatment through the end of treatment.

### Statistical analysis

Approximately 80 patients with nonlymphomatous cancer were planned to be enrolled, which was considered adequate to detect the antitumor activity of brentuximab vedotin in CD30-expressing disease. The efficacy-evaluable set of patients included all treated patients who had undergone a baseline disease assessment and at least 1 evaluable post-baseline assessment, and was used in analysis of efficacy. The all-treated patients set included all patients who had received at least 1 dose of brentuximab vedotin, and was used in the analysis of safety. All statistical analyses were performed using SAS® software.

The primary endpoint of the study was the objective response rate (ORR), defined as the proportion of subjects with complete response/remission (CR) or partial response/remission (PR). The ORR and its two-sided 95% confidence interval (CI) were calculated using the Clopper-Pearson method [24].

Secondary endpoints included progression-free survival (PFS), rate of CR, duration of ORR, duration of CR, safety outcomes, and estimates of selected PK parameters. Progression-free survival, duration of response, and duration of CR were analyzed using Kaplan-Meier methodology. Median duration of response, median PFS, and median duration of CR and their 95% CIs were calculated using the complementary log-log transformation method [25]. Complete response rate was calculated with its 95% CI using the Clopper-Pearson method [24].

All safety endpoints were summarized using the all-treated patients set. Adverse events (AEs) were coded using the Medical Dictionary for Regulatory Activities version 18.0. Treatment-emergent AEs were defined as newly occurring or worsening events following the first dose of brentuximab vedotin. Laboratory values were graded according to the National Cancer Institute Common Terminology Criteria for Adverse Events, version 4.03.

## Results

### Patients

**Fig. 1:**
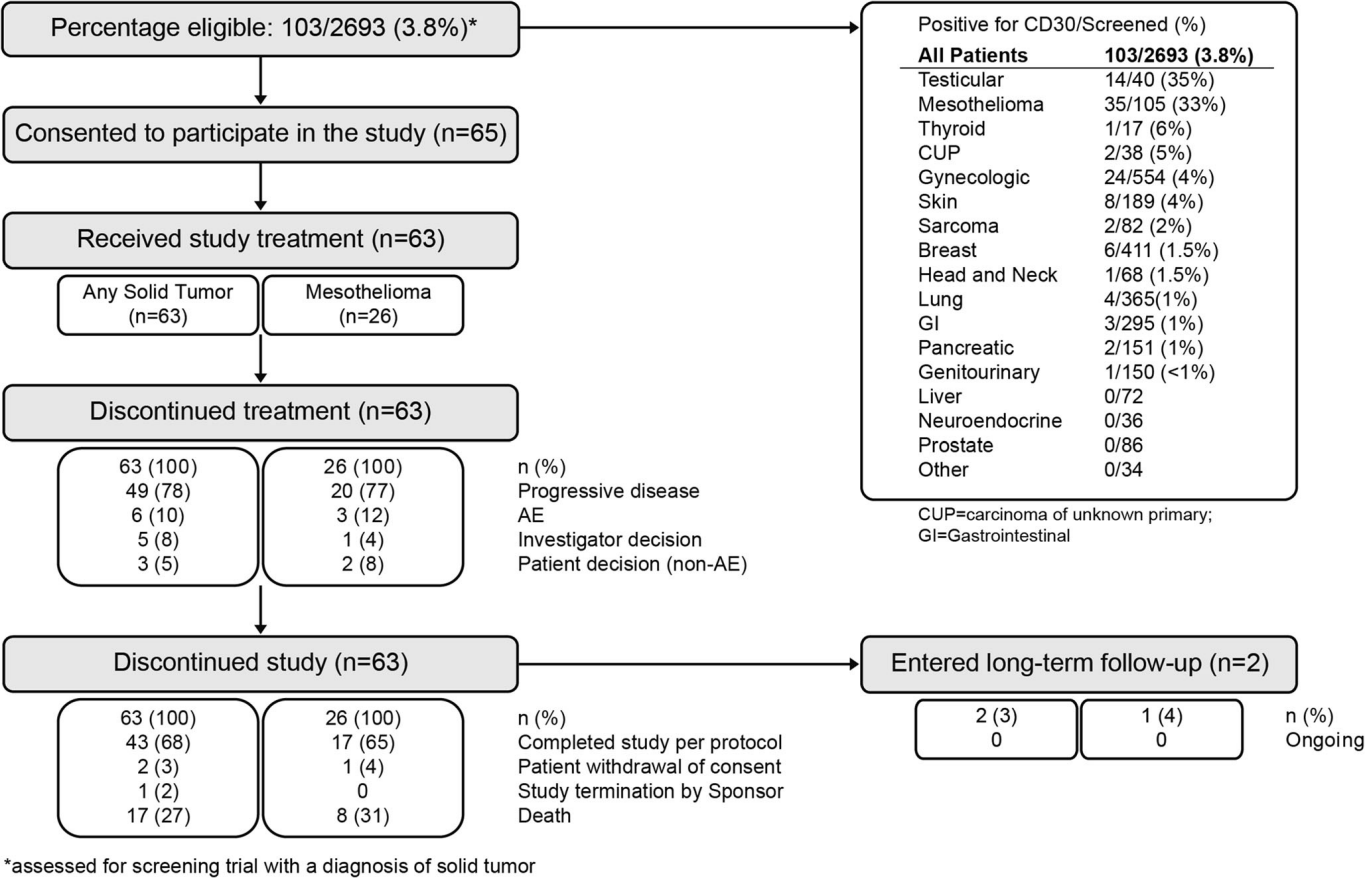
Patient disposition (Solid Tumors)

A total of 2693 patients with solid tumors were screened for CD30 expression in a companion screening protocol (SGN00–001), 103 (3.8%) of whom screened positive for CD30 (Fig. 1). From that population, a total of 63 patients with CD30-expressing solid tumors consented and were treated in this study (83 patients treated overall). The median age of all patients with solid tumors was 64 years (range, 24 to 85) (Table 1). In general, baseline demographic characteristics of all patients with solid tumors were similar between dose groups. Most patients (92% overall) had been previously treated with systemic therapies. Among patients who had received prior systemic therapy, the median number of prior systemic regimens was 2 to 3 (median = 2.5; range, 1 to 13), and most patients had achieved a best response of SD or PD (20 patients [32%] each) on their most recent regimen. The median percent of malignant cells expressing CD30 in the pathology specimens of all patients with solid tumors was 40% with a wide range (range, 10% to 100%).

**Table 1:** Patient demographic and disease characteristics (all-treated patient set)

	All patients with solid tumors			Patients with mesothelioma		
	1.8 mg/kg (*N* = 40)	2.4 mg/kg (*N* = 23)	Total (*N* = 63)	1.8 mg/kg (*N* = 10)	2.4 mg/kg (*N* = 16)	Total (*N* = 26)
Median age in years (range)	63.5 (24, 85)	65.0 (28, 85)	64.0 (24, 85)	68.5 (52, 85)	67.0 (42, 81)	67.0 (42, 85)
Sex, n (%)						
Male	21 (53)	13 (57)	34 (54)	8 (80)	11 (69)	19 (73)
Female	19 (48)	10 (43)	29 (46)	2 (20)	5 (31)	7 (27)
Race, n (%)						
Asian	1 (3)	0	1 (2)	0	0	0
Black or African American	2 (5)	0	2 (3)	0	0	0
White	37 (93)	22 (96)	59 (94)	10 (100)	16 (100)	26 (100)
Other	0	1 (4)	1 (2)	0	0	0
Eastern Cooperative Oncology Group performance status, n (%)						
0	12 (30)	3 (13)	15 (24)	2 (20)	1 (6)	3 (12)
1	16 (40)	12 (52)	28 (44)	5 (50)	9 (56)	14 (54)
Missing	12 (30)	8 (35)	20 (32)	3 (30)	6 (38)	9 (35)
Tumor type, n (%)						
Pleural Mesothelioma	6 (15)	15 (65)	21 (33)	6 (60)	15 (94)	21 (81)
Peritoneal Mesothelioma	4 (10)	1 (4)	5 (8)	4 (40)	1 (6)	5 (19)
Other^a^	30 (75)	7 (30)	37 (59)	NA	NA	NA
Median percent positivity of CD30 (range)	42.5 (10, 100)	30.0 (10, 95)	40.0 (10, 100)	50.0 (10, 75)	30.0 (10, 95)	40.0 (10, 95)
Prior systemic regimens, n	35	23	58	7	16	23
Median number	3.0	2.0	2.5	2.0	2.0	2.0
Best response on prior regimen						
Complete Remission	0	1 (4)	1 (2)	0	0	0
Partial Remission	5 (13)	1 (4)	6 (10)	0	0	0
Stable Disease	12 (30)	8 (35)	20 (32)	3 (30)	6 (38)	9 (35)
Progressive Disease	13 (33)	7 (30)	20 (32)	3 (30)	5 (31)	8 (31)
Unknown/Other	10 (25)	6 (26)	16 (25)	4 (40)	5 (31)	9 (35)
Time from initial diagnosis to first dose, n	38	23	61	10	16	26
Median in months	25.2	15.4	20.1	13.1	16.6	15.3

*NA* not applicable

^a^Includes breast - ductal - triple negative; carcinoma of unknown primary; gastrointestinal – anal; genitourinary - other (genitourinary) - urethral squamous cell carcinoma; gynecologic - endometrial adenocarcinoma; gynecologic - ovarian, epithelial; gynecologic - ovarian, other - small cell ovarian cancer, neurodendocrine type, hypercalcemic type; lung - small cell - unknown (lung); other: carcinoma of the appendix; pancreatic - other (pancreatic) - undifferentiated carcinoma with osteoclast-like giant cells; sarcoma – rhabdomyosarcoma; skin – melanoma; skin – squamous cell carcinoma; testicular - germ cell tumors; testicular - other (testicular) – leydig; testicular - other (testicular) – sertoli; and thyroid - other (thyroid) – anaplastic

The most common disease subtypes among patients with CD30-positive solid tumors were testicular cancer, which has already been reported [16], and mesothelioma. Twenty-six patients who were treated on this study had mesothelioma; 21 patients had malignant pleural mesothelioma [MPM] and 5 patients had peritoneal disease. Demographic characteristics of patients with mesothelioma were similar to those of all solid tumor patients (Table 1).

Among patients with mesothelioma, those with MPM tended to be older (median age 72 years [range, 42 to 85]) than patients with peritoneal disease (median age 59 years [range, 51 to 65]), with shorter median time from initial pathologic diagnosis to first dose of brentuximab vedotin (13.3 months versus 60.7 months) and higher median CD30 expression level (50% versus 10%). Among mesothelioma patients who had received prior systemic therapy, patients with MPM had received a median of 2 (range, 1 to 4) prior regimens while those with peritoneal disease had received a median of 1 (range, 1 to 9) prior regimen. All but 3 patients with MPM had received prior systemic cancer therapy; all with prior systemic therapy had received prior cisplatin or carboplatin, and all but 1 had received pemetrexed.

Most patients (68%) completed the study per the protocol (Fig. 1); the most common reason for study discontinuation other than study completion was death (27%). The most common reason for discontinuation of brentuximab vedotin in any dose group or disease subset was progressive disease (78% overall). No subjects are currently in long term follow-up.

### Efficacy

**Fig. 2:**
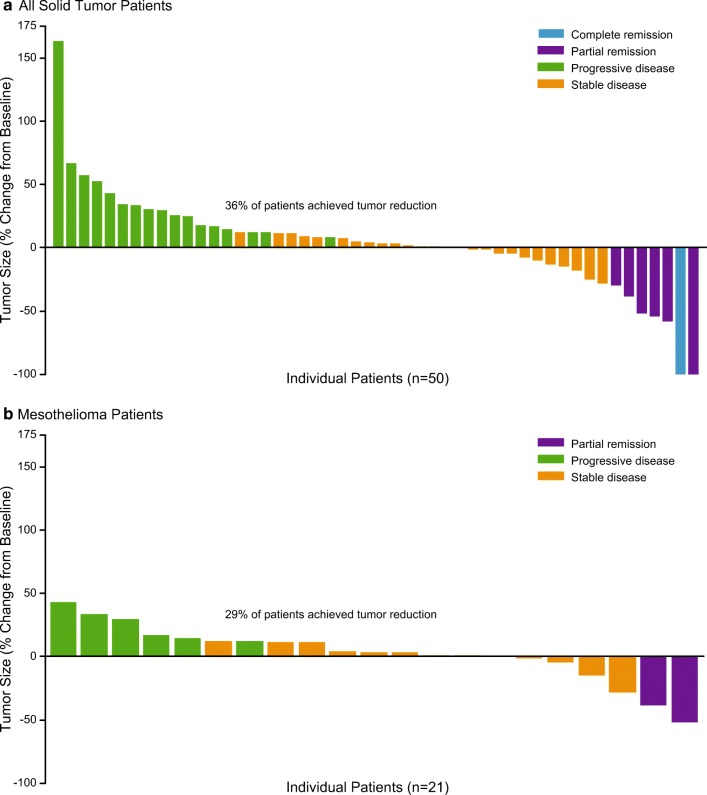
Change in target lesion size after treatment with brentuximab vedotin for all solid tumor patients (**a**) and mesothelioma patients (**b**)

Fifty-nine patients with solid tumors were evaluable for response, including 23 patients with mesothelioma. The ORR in all patients with solid tumors was 11% (95% CI 4.9, 22.9), and was numerically higher for patients in the 2.4 mg/kg dose group (18% [95% CI 5.2, 40.3]) than in the 1.8 mg/kg dose group (8% [95% CI 1.7, 21.9]) (Table 2). The disease control rate (DCR), defined as achieving stable disease or better at any point in the study, was 55% (95% CI 42.4, 68.8). An objective response was observed in 7 patients (12%, 1 CR and 6 PRs). Of these, the testicular and mesothelioma subtypes had more than one patient with a response, including the CR patient with a diagnosis of testicular germ cell tumor. The median duration of objective response among all solid tumor patients was 2.92 months (95% CI: 1.51 to 7.03 months) (Table 2). Overall, 36% (18/50) of patients with at least one postbaseline assessment achieved a reduction in tumor size after treatment with brentuximab vedotin, including 1 patient with a CR and 1 patient with a PR who achieved almost 100% reduction in the size of the target lesion (Fig. 2a).

**Table 2:** Summary of best response (efficacy-evaluable patient set)

	All patients with solid tumors			Patients with mesothelioma		
	Initial dose ≤ 1.8 mg/kg (*N* = 37)	Initial dose 2.4 mg/kg (*N* = 22)	Total (*N* = 59)	Initial dose ≤ 1.8 mg/kg (*N* = 8)	Initial dose 2.4 mg/kg (*N* = 15)	Total (*N* = 23)
Best response^a,b^, n (%)						
CR	1 (2)	0	1 (1)	0	0	0
PR	2 (5)	4 (18)	6 (10)	0	2 (13)	2 (8)
SD	18 (48)	8 (36)	26 (44)	6 (75)	7 (46)	13 (56)
Progression	16 (43)	10 (45)	26 (44)	2 (25)	6 (40)	8 (34)
PD	12(32)	6 (27)	18 (31)	1 (12)	4 (26)	5 (21)
CP^c^	4 (10)	4 (18)	8 (14)	1 (12)	2 (13)	3 (13)
ORR, n (%)	3 (8)	4 (18)	7 (11)	0	2 (13)	2 (8)
95% CI^d^	(1.7, 21.9)	(5.2, 40.3)	(4.9, 22.9)	(0.0, 36.9)	(1.7, 40.5)	(1.1, 28.0)
Disease control rate, n (%)	21 (56)	12 (54)	33 (55)	6 (75)	9 (60)	15 (65)
95% CI^d^	(39.5, 72.9)	(32.2, 75.6)	(42.4, 68.8)	(34.9, 96.8)	(32.3, 83.7)	(42.7, 83.6)
Patients with PD or death, n (%)	37 (93)	21 (91)	58 (92)	10 (100)	15 (94)	25 (96)
Median PFS, months (95% CI^a^)	2.0 (1.2, 3.0)	2.1 (1.1, 2.8)	2.1 (1.2, 2.8)	2.5 (0.3, 8.2)	2.1 (1.0,2.8)	2.5 (1.2,2.8)
Patients with objective response, n (%)	3 (8)	4 (18)	7 (11)	0	2 (13)	2 (8)
Median objective response, months (95% CI^e^)	6.18 (1.84, 23.46)	2.63 (1.51, 7.03)	2.92 (1.51, 7.03)	–	2.63 (2.33, 2.92)	2.63 (2.33, 2.92)
Patients with CR, n (%)	1 (2)	0	1 (1)	0	0	0
Median CR, months (95% CI^e^)	22.31 (−, −)	–	22.31 (−, −)	–	–	–

Complete response was observed for one patient with testicular cancer. Partial responses were observed for the following 5 tumor types (6 patients overall): mesothelioma (2 patients), testicular (1 patient), breast (1 patient), pancreatic - other (pancreatic) - undifferentiated carcinoma with osteoclast-like giant cells (1 patient); and carcinoma of unknown primary (1 patient)

*Abbreviations*: *CI* confidence interval, *CP* clinical progression, *CR* complete response, *DCR* disease control rate (CR + SD + CR), *ORR* overall response rate (CR + PR), *PD* progressive disease, *PR* partial response, *RECIST* Response Evaluation Criteria in Solid Tumors, *SD* stable disease; −- = not applicable

^a^Best response per RECIST 1.1

^b^CR, PR, SD, PD, CP, and Not evaluable are mutually exclusive. Patients with both PD and CP were counted as PD

^c^Symptomatic deterioration with no RECIST evaluation and progression indicated as reason off treatment

^d^Two-sided 95% exact CI, computed using the Clopper-Pearson method [24]

^e^Computed using the method of Collet

Among the 23 evaluable patients with mesothelioma, 19 had MPM and 4 had peritoneal disease. No CRs were observed. The ORR was 8% (95% CI 1.1, 28.0), and included 2 patients (13%) with MPM treated with 2.4 mg/kg brentuximab vedotin Q3W (Table 2). The median duration of objective response in these 2 patients was 2.63 months. No patients with MPM treated with 1.8 mg/kg brentuximab vedotin and no patients with peritoneal mesothelioma achieved a PR. The overall DCR in mesothelioma patients was 65% (95% CI 42.7, 83.6). Overall, 29% of patients with mesothelioma achieved a reduction in the size of their target lesion following treatment with brentuximab vedotin (Fig. 2b). The 2 patients with mesothelioma who achieved a PR during the study had reductions in tumor size of approximately 50% and 40%.

**Fig. 3:**
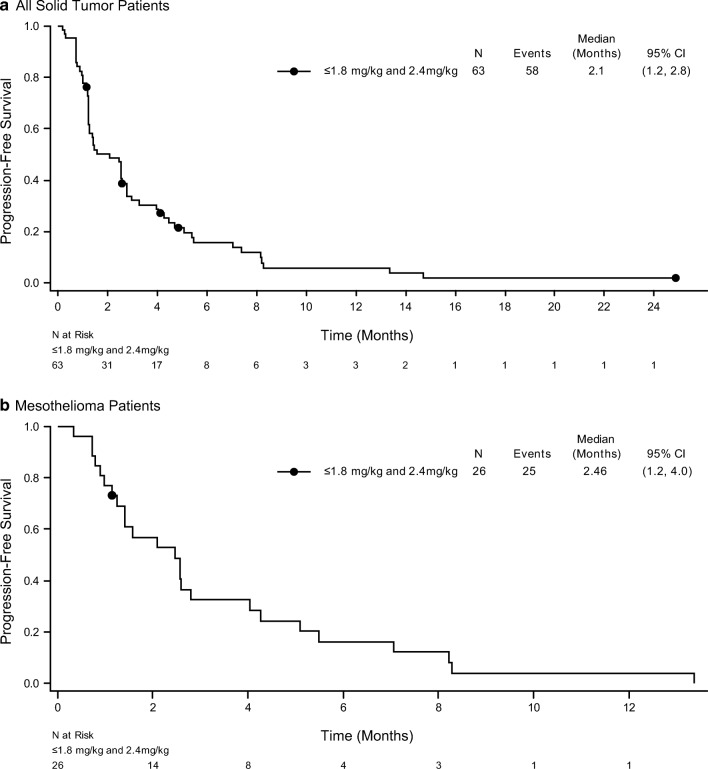
Progression-free survival was assessed for all solid tumor patients (**a**) and mesothelioma patients (**b**)

Most patients with solid tumors (92% overall) experienced progressive disease or death during the study. The median PFS was 2.1 months (95% CI: 1.2 to 2.8 months) overall (Fig. 3a) and there were no dose-related trends. The median PFS in patients with mesothelioma was 2.5 months (95% CI 1.2, 4.0) (Fig. 3b).

### Adverse events

In all treated solid tumor patients, the median duration of brentuximab vedotin exposure was 6.1 weeks and the median number of treatment cycles per patient was 2.0. There were no differences in median duration of exposure for patients receiving an initial dose of 1.8 mg/kg Q3W compared with 2.4 mg/kg Q3W. Patients with mesothelioma had a median duration of treatment of 8 weeks and the median number of treatment cycles per patient was 2.5. Within this treatment group, the median duration of exposure for patients receiving an initial dose of 1.8 mg/kg Q3W was 11.4 weeks, compared with 6.6 weeks for patients who received an initial dose of 2.4 mg/kg Q3W brentuximab vedotin. Patients with peritoneal mesothelioma had a median duration of treatment of 9 weeks and the median number of treatment cycles per patients was 3. Within this treatment group, the median duration of exposure for patients receiving an initial dose of 1.8 mg/kg Q3W was 21.1 weeks, compared with 9 weeks for patients who received an initial dose of 2.4 mg/kg Q3W brentuximab vedotin.

Most patients (98% overall) reported at least 1 AE during the study period. Adverse events occurring in at least 10% of all patients are presented overall and by dose in Supplemental Table 1. The most common AEs (>30%) among all patients included fatigue (57%), nausea (33%), and decreased appetite (32%). Other than a higher incidence of peripheral sensory neuropathy in the 2.4 mg/kg dose group, described below, there were no notable differences in AEs between dose groups. Overall, 73% of patients had AEs considered by the investigator to be related to treatment with brentuximab vedotin. The most common treatment-related events (>20% overall) included fatigue (38%), nausea (24%), alopecia (21%), and peripheral sensory neuropathy (21%).

Six patients (10%) overall discontinued study treatment due to an AE, including fatigue (2 patients [3%]), acute kidney injury, erythroleukemia, respiratory failure, and sepsis (1 patient [2%] each). There were no differences in the incidence or type of AEs leading to treatment discontinuation between dose groups.

A total of 43% of patients reported at least 1 serious adverse event (SAE) during the study period (Supplemental Table 2). The most common (≥5%) SAEs not related to disease progression among all patients were abdominal pain (6%); and dyspnea, hypoxia, and respiratory failure (5% each). The incidence and pattern of SAEs were similar regardless of the initial dose of brentuximab vedotin received.

The majority of all patients (60%) had at least 1 Grade 3 or higher AE (Supplemental Table 3). The only Grade 3 or higher AE occurring in >10% of patients was fatigue (16%). The incidence and pattern of Grade 3 or higher AEs was similar between dose groups.

Neuropathy-related events that occurred during the study included peripheral sensory neuropathy (19%) and peripheral motor neuropathy (10%). Most events of neuropathy were Grade 1 or 2 in severity; 2 (3%) patients each reported Grade 3 or greater events of peripheral motor neuropathy and peripheral sensory neuropathy. Peripheral sensory neuropathy was observed more frequently in the 2.4 mg/kg brentuximab vedotin dose group (26%) than in the 1.8 mg/kg dose group (15%). No neuropathy events were serious and none led to treatment discontinuation.

Among patients with mesothelioma, the most frequently observed AEs did not differ from those observed in all solid tumor patients. Serious AEs were observed in 12 patients with mesothelioma, the most frequent of which included events related to the patients underlying disease (malignant mesothelioma [15%], dyspnea [12%], hypoxia [8%], and respiratory failure [8%]).

## Discussion

This phase 2 trial is the first to report a 3.8% incidence of CD30 in solid tumors using a 10% positivity cutoff. Tumor screenings for antigens are becoming increasingly common, and this data adds to a previous report listing CD30 incidence according to primary solid tumor site [26]. This trial also demonstrated an ORR of 11% among 59 efficacy-evaluable patients with solid tumors, which is similar to already approved second-line treatments. One patient had a CR and 6 patients had PR. The patient who achieved a CR during the study had a testicular germ cell tumor and was treated with 1.8 mg/kg brentuximab vedotin. A full description of this patient’s clinical course has been described previously [16]. Although the ORR in this study was only 11%, 36% of all solid tumor patients achieved a reduction in their tumor size from baseline. Overall, median PFS was 2.1 months and no dose-related trends in PFS were observed between the two dose groups.

Adverse events were common and were observed at similar frequencies regardless of dose. The most common AEs included fatigue, nausea, and loss of appetite. Consistent with the known safety profile of brentuximab vedotin, the most common related AEs were fatigue, nausea, alopecia, and sensory peripheral neuropathy. Neuropathy events observed in this study, including peripheral sensory and motor neuropathies, were generally Grade 1 to 2 in severity. None were considered serious or led to treatment discontinuation. Peripheral sensory neuropathy was observed more frequently in the higher brentuximab vedotin dose cohort. As MMAE, the cytotoxic component of brentuximab vedotin, is an antitubulin agent, this is consistent with the known class effects of microtubule inhibitors [27, 28].

Nearly all patients with malignant pleural mesothelioma progress during or after first-line treatment. Acceptable second-line therapy options include single-agents, which are associated with low response rates (<20%) and ~50% DCR [29–31]. In the current study, the 21 patients with MPM treated with brentuximab vedotin achieved a DCR of 63%. The observed ORR and DCR in these patients were comparable to those reported in other prospective studies of single agents such as vinorelbine, gemcitabine, or PD-1/PD-L1 inhibitors for pleural mesothelioma patients [32, 33].

Due to the highly active nature of brentuximab vedotin in lymphomatous cancers, study of this drug in CD30-expressing nonlymphomatous malignancies was warranted. The safety profile of brentuximab vedotin in patients with solid tumors was similar the known safety profile of brentuximab vedotin administered at the approved dose level of 1.8 mg/kg Q3W in patients with lymphoma. The results of this analysis suggest brentuximab vedotin had modest single agent activity in CD30-positive solid tumors.

## Electronic supplementary material

ESM 1 (DOCX 19 kb)
