# Supplementary material for: A phase 2, open-label study of brentuximab vedotin in patients with CD30-expressing solid tumors
Source: Invest New Drugs. 2019 Apr 16;37(4):738–47. doi: 10.1007/s10637-019-00768-6 (PMC6647393; doi:10.1007/s10637-019-00768-6)
Supplement: Supplementary file 1 — (DOCX 19 kb) [file 10637_2019_768_MOESM1_ESM.docx]

Supplemental Table 1 Adverse Events Occurring in ≥10% of All Patients (All-Treated Patient Set)

|  | **1.8 mg/kg (N=40)** | **2.4 mg/kg (N=23)** | **Total (N=63)** |
| --- | --- | --- | --- |
| Any adverse event^a^ | 39 (98) | 23 (100) | 62 (98) |
| Fatigue | 25 (63) | 11 (48) | 36 (57) |
| Nausea | 11 (28) | 10 (43) | 21 (33) |
| Decreased appetite | 11 (28) | 9 (39) | 20 (32) |
| Diarrhoea | 12 (30) | 6 (26) | 18 (29) |
| Constipation | 9 (23) | 7 (30) | 16 (25) |
| Dyspnoea | 9 (23) | 7 (30) | 16 (25) |
| Vomiting | 10 (25) | 6 (26) | 16 (25) |
| Alopecia | 6 (15) | 7 (30) | 13 (21) |
| Peripheral sensory neuropathy | 6 (15) | 6 (26) | 12 (19) |
| Pruritus | 6 (15) | 6 (26) | 12 (19) |
| Rash | 8 (20) | 3 (13) | 11 (17) |
| Abdominal pain | 6 (15) | 4 (17) | 10 (16) |
| Pyrexia | 6 (15) | 4 (17) | 10 (16) |
| Dizziness | 4 (10) | 5 (22) | 9 (14) |
| Back pain | 4 (10) | 4 (17) | 8 (13) |
| Dehydration | 5 (13) | 3 (13) | 8 (13) |
| Chills | 5 (13) | 2 (9) | 7 (11) |
| Cough | 4 (10) | 3 (13) | 7 (11) |
| Insomnia | 3 (8) | 4 (17) | 7 (11) |
| Headache | 4 (10) | 2 (9) | 6 (10) |
| Oedema peripheral | 2 (5) | 4 (17) | 6 (10) |
| Peripheral motor neuropathy | 4 (10) | 2 (9) | 6 (10) |

1. Treatment-emergent adverse events are presented and defined as newly occurring (not present at Baseline) or worsening after first dose of brentuximab vedotin.

Supplemental Table 2 Serious Adverse Events Occurring in >1 Patient (All-Treated Patient Set)

|  | **1.8 mg/kg (N=40)** | **2.4 mg/kg (N=23)** | **Total (N=63)** |
| --- | --- | --- | --- |
| Any serious adverse event | 18 (45) | 9 (39) | 27 (43) |
| Abdominal pain | 2 (5) | 2 (9) | 4 (6) |
| Mesothelioma malignant | 2 (5) | 2 (9) | 4 (6) |
| Dyspnoea | 2 (5) | 1 (4) | 3 (5) |
| Hypoxia | 3 (8) | 0 | 3 (5) |
| Respiratory failure | 2 (5) | 1 (4) | 3 (5) |
| Acute kidney injury | 2 (5) | 0 | 2 (3) |
| Ovarian cancer | 1 (3) | 1 (4) | 2 (3) |
| Pleural effusion | 2 (5) | 0 | 2 (3) |
| Sepsis | 2 (5) | 0 | 2 (3) |
| Small intestinal obstruction | 1 (3) | 1 (4) | 2 (3) |
| Urinary tract infection | 1 (3) | 1 (4) | 2 (3) |

Supplemental Table 3 Grade 3 or Higher Adverse Events Occurring in >1 Patient (All-Treated Patient Set)

|  | **1.8 mg/kg (N=40)** | **2.4 mg/kg (N=23)** | **Total (N=63)** |
| --- | --- | --- | --- |
| Any Grade 3 or higher adverse event^a^ | 25 (63) | 13 (57) | 38 (60) |
| Fatigue | 5 (13) | 5 (22) | 10 (16) |
| Abdominal pain | 2 (5) | 2 (9) | 4 (6) |
| Dyspnoea | 2 (5) | 2 (9) | 4 (6) |
| Mesothelioma malignant | 2 (5) | 2 (9) | 4 (6) |
| Muscular weakness | 2 (5) | 1 (4) | 3 (5) |
| Respiratory failure | 2 (5) | 1 (4) | 3 (5) |
| Sepsis | 3 (8) | 0 | 3 (5) |
| Acute kidney injury | 2 (5) | 0 | 2 (3) |
| Ascites | 1 (3) | 1 (4) | 2 (3) |
| Diverticulitis | 1 (3) | 1 (4) | 2 (3) |
| Hypoxia | 2 (5) | 0 | 2 (3) |
| Neutropenia | 1 (3) | 1 (4) | 2 (3) |
| Ovarian cancer | 1 (3) | 1 (4) | 2 (3) |
| Peripheral motor neuropathy | 1 (3) | 1 (4) | 2 (3) |
| Peripheral sensory neuropathy | 1 (3) | 1 (4) | 2 (3) |
| Pleural effusion | 2 (5) | 0 | 2 (3) |
| Rash | 2 (5) | 0 | 2 (3) |
| Small intestinal obstruction | 1 (3) | 1 (4) | 2 (3) |
| Urinary tract infection | 1 (3) | 1 (4) | 2 (3) |

1. Grade 3 or higher treatment-emergent adverse events are presented and defined as newly occurring (not present at Baseline) or worsening after first dose of brentuximab vedotin.
